# A Potential Nutritional Indicator Predictable for Stroke-Related Sarcopenia

**DOI:** 10.31662/jmaj.2021-0206

**Published:** 2021-12-28

**Authors:** Yoshihide Sunada

**Affiliations:** 1 Department of Neurology, Kawasaki Medical School, Kurashiki, Japan

**Keywords:** sarcopenia, nutritional indicator, stroke

Sarcopenia was originally proposed by Rosenberg in 1989 ^(1)^ as a concept that means age-related loss of muscle mass. Thereafter, the concept expanded with the spread of this term. According to the consensus statement of European Working Group on Sarcopenia in Older People, sarcopenia is classified into primary (age-related) sarcopenia, which has no evident cause other than aging, and secondary sarcopenia, which has apparent causes such as immobility, malnutrition, and underlying diseases ^(2)^. After the onset of hemiparalytic stroke, muscle atrophy occurs due to various factors, such as denervation, disuse, spasticity, remodeling, and inflammation, and has recently been categorized as stroke-related sarcopenia.

Since undernutrition underlies the pathophysiology of sarcopenia, it is reasonable to speculate that the nutritional status of patients with stroke will have a significant impact on the prognosis of stroke-related sarcopenia. In fact, there are increasing reports that nutritional status is involved in functional and performance prognosis in patients in the subacute and convalescent stages of stroke. In addition, undernutrition even in the prestroke or acute phase of stroke is reported to be a poor prognosis factor ^(3)^. Although no consensus has yet been achieved on nutritional assessment tools in stroke patients, Nutritional Risk Screening (NRS) 2002, Subjective Global Assessment, Mini Nutritional Assessment (MNA) and MNA-Short Form (MNA-SF) are often used, including in the acute stage. To date it has been elusive which is optimal among several nutritional indicators for predicting the severity of secondary sarcopenia during acute stage in patients with stroke.

In this issue of *JMA Journal*, Kokura et al. report potential usefulness of MNA-SF to predict muscle atrophy of nonparalytic limbs in acute stroke patients ^(4)^. Their retrospective cohort study evaluated the capability of three nutritional indicators, Geriatric Nutritional Risk Index (GNRI), Controlling Nutritional Status (CONUT), MNA-SF to predict changes in quadriceps muscle thickness (QMT) in 2 weeks from admission using ultrasound imaging in 118 patients with the acute stage of hemiparalytic stroke. It is noteworthy that among several surrogate markers for skeletal muscle function, QMT is closely associated with poststroke physical activity ^(5)^, as quadriceps muscle plays important roles in independent standing and gait. They found a significant difference of QMT changes in the nonparalytic side between malnourished patients and normal nutritional status patients categorized by MNA-SF, whereas no significant difference was detected between the groups stratified based on either GNRI or CONUT. Interestingly, the MNA-SF score was not independently associated with QMT changes in the paralytic limbs. QMT in paralyzed limbs may be affected by other factors including immobility and spasticity beside the nutritional status.

GNRI and CONUT scores are based on blood biomarkers such as serum albumin, total cholesterol, and total lymphocyte count in addition to body weight. In patients with stroke GNRI and CONUT scores were independent predictive factors of mortality and functional prognosis. However this study showed that they are insufficient for predicting changes in the muscle mass. In contrast MNA-SF evaluates nutritional risk without use of biomarkers. Instead, it is scored in six domains including BMI, weight loss, appetite, mobility, stress/acute disease, and cognitive function. Therefore, MNA-SF could predict changes in muscle mass caused by insufficient food intake and immobility.

Further, it is intriguing to know the relationship between the QMT changes and functional outcome in hemiparalytic stroke patients. In this study they also performed the univariate analysis of gait independence degree at discharge assessed by using the gait score of the Functional Independence Measure tool. There was a significant difference in the 2 week changes in QMT in the nonparalytic limb between the independent and nonindependent groups. The result indicates that maintaining muscle mass in nonparalyzed limbs during the acute stage of stroke is associated with reacquisition of gait independence. Conclusively, the significance of this paper is that MNA-SF is useful as a nutritional evaluation tool for predicting QMT changes of nonparalyzed limbs during the acute stage of stroke, which is a potential functional prognostic factor.

As the authors described, one of the limitations of this study is that it is a retrospective cohort study conducted as a post-hoc analysis of a previous prospective study in a single hospital. Larger multicenter prospective studies are required to confirm current conclusions. However, this study provides the initial findings on early changes of muscle mass after stroke using ultrasound and the capability of nutritional indicators for prediction of muscle atrophy. This may contribute to proper nutritional care of stroke patients especially at risk of malnutrition to prevent stroke-related sarcopenia and to improve functional outcome.

## Article Information

### Conflicts of Interest

None
